# Innovative modification strategies and emerging applications of natural hydrogel scaffolds for osteoporotic bone defect regeneration

**DOI:** 10.3389/fbioe.2025.1591896

**Published:** 2025-04-28

**Authors:** Yanan Chen, Qinghua Zhao

**Affiliations:** School of Medical Instrument and Food Engineering, University of Shanghai for Science and Technology, Shanghai, China

**Keywords:** natural hydrogel, hydrogel scaffold, osteoporosis, bone defect, bone repair

## Abstract

Osteoporosis, a prevalent systemic metabolic bone disease, is characterized by diminished bone mass, microarchitectural deterioration of bone tissue, and heightened bone fragility. In osteoporotic patients, chronic and progressive bone loss often leads to fractures and, in advanced cases, critical-sized bone defects. While traditional bone repair approaches are constrained by significant limitations, the advent of bioactive scaffolds has transformed the therapeutic paradigm for osteoporotic bone regeneration. Among these innovations, natural polymer-based hydrogel scaffolds have emerged as a particularly promising solution in bone tissue engineering, owing to their superior biocompatibility, tunable biodegradation properties, and exceptional ability to replicate the native extracellular matrix environment. This review systematically explores recent breakthroughs in modification techniques and therapeutic applications of natural hydrogel scaffolds for osteoporotic bone defect repair, while critically analyzing existing clinical challenges and proposing future research trajectories in this rapidly evolving field.

## 1 Introduction

Osteoporosis (OP) is a systemic metabolic bone disorder characterized by reduced bone mass and microstructural deterioration (Compston et al., 2019). Prolonged and progressive bone loss in OP patients often leads to the development of fractures and, in severe cases, bone defects (Li et al., 2023). OP predominantly affects individuals over 50 years of age, with a particularly high prevalence among middle-aged and elderly women (Wang J. et al., 2023). As the global population ages, the incidence of osteoporosis and its associated bone defects is rising significantly, imposing a substantial burden on both individuals and society. Traditional treatments for OP-related bone defects, including pharmacological therapies and surgical interventions, can provide symptomatic relief to some extent. However, these approaches are often limited by issues such as adverse drug effects and surgical complications, highlighting the need for more effective and safer therapeutic strategies (Manoochehri et al., 2022; Deininger et al., 2021).

In recent years, tissue engineering technologies offer innovative solutions to addressing bone defects (Wang et al., 2024a; Wang et al., 2023b; Wang et al., 2021a; Hamza et al., 2025). Compared with traditional bone graft materials (e.g., autogenous bone, allograft bone), hydrogel has better biocompatibility and plasticity, and better adapts to the complex morphology of bone defects (Hamza et al., 2025; Wang et al., 2024b). Among various biomaterials, natural hydrogels stand out in bone tissue engineering due to their unique physicochemical and biological properties (Guo et al., 2021). They are highly biocompatible, degradable, and mimic the natural extracellular matrix (ECM), creating an ideal environment for cell adhesion, proliferation, and differentiation, which promotes tissue regeneration (Liao et al., 2021; Abou-Okeil et al., 2024; Ho et al., 2022). Additionally, natural hydrogels can act as effective carriers for localized and sustained delivery of therapeutic agents, such as drugs and growth factors, to improve bone repair and regeneration (Ai et al., 2023). Compared to traditional systemic drug delivery, this local delivery system significantly improves drug utilisation and reduces systemic side effects.

While numerous reviews have explored the applications of natural hydrogels in bone defect regeneration, there remains a significant gap in comprehensive analyses specifically addressing osteoporotic bone defects (Ding et al., 2023; El-Rashidy et al., 2017; Li et al., 2025). The unique mechanical properties, cellular functions, and pathological bone microenvironment associated with osteoporosis necessitate distinct modification and application strategies, which have not been systematically reviewed to date. By way of example, researchers have developed reinforced hydrogel composites to improve the low mechanical strength of osteoporotic bone defects and designed smart-responsive hydrogels to combat chronic inflammation. However, clinical translation of these strategies faces challenges, including material long-term stability, biosafety, and scalable production feasibility.

This review aims to provide a comprehensive analysis of natural hydrogel applications in osteoporotic bone defect treatment, with particular emphasis on two critical aspects: innovative modification strategies tailored to the specific requirements of osteoporotic bone regeneration, and diverse application approaches, such as bioactive scaffold design and advanced drug delivery systems. Furthermore, we critically examine current research limitations and challenges, while proposing future directions for the development of natural hydrogel-based therapies in this specialized field.

## 2 Pathologic features of osteoporotic bone defects and requirements for repair materials

### 2.1 Pathologic features of osteoporotic bone defects

The development of osteoporotic bone defects is a complex, multifactorial process involving multiple pathological stages. At the macroscopic level, the bone matrix at osteoporotic defect sites exhibits significantly reduced mechanical strength, characterized by decreased bone density and microstructural deterioration. This decline in mechanical properties results in increased bone brittleness, making the bone incapable of providing adequate mechanical support. Consequently, the risk of internal fixation failure is markedly elevated (Hemmeler et al., 2017; Reichert et al., 2009). Furthermore, conventional restorative materials often exhibit a mechanical mismatch with the osteoporotic bone matrix, leading to potential issues such as stress shielding or mechanical failure during treatment (Egermann et al., 2005).

Osteoporosis development fundamentally involves disrupted bone remodeling homeostasis, characterized by excessive osteoclastic activity and insufficient osteoblastic function (Wang LT. et al., 2023). This imbalance is regulated by a complex network of signaling pathways and cytokines that govern bone metabolism. The ongoing cycle of osteoclast overactivity and osteoblast suppression weakens bones and impairs their natural repair ability (Colnot, 2005). Additionally, bone marrow mesenchymal stem cells (BMSCs), essential for bone regeneration, are fewer in number and show reduced proliferation and differentiation in osteoporosis patients, further complicating bone defect repair (Colnot, 2005).

In addition to bone tissue changes, the compromised bone repair microenvironment also plays a critical role in the progression of osteoporotic bone defects. Bone regeneration relies heavily on angiogenesis, but osteoporotic sites often lack sufficient blood supply (Ding et al., 2011). This not only hampers stem cell recruitment and osteogenic differentiation, thereby delaying bone healing, but also complicates the reconstruction of the vascular network, increasing the risks of infection and nonunion (Brandi and Collin-Osdoby, 2006). Additionally, osteoporotic bone defects are frequently accompanied by a chronic inflammatory response (Stout and Suttles, 2005). The infiltration of immune cells and the overexpression of pro-inflammatory cytokines in this inflammatory microenvironment disrupt the bone repair process. Specifically, chronic inflammation inhibits osteoblast differentiation and function while promoting osteoclast activation, creating a hostile environment that severely impedes bone regeneration and repair (Eming et al., 2017; Jackaman et al., 2017). Moreover, osteoclasts release excess H^+^, acidifying the bone microenvironment and further hindering bone repair (Fu et al., 2022).

In summary, the pathological features of osteoporotic bone defects encompass both structural and biological alterations, including reduced mechanical strength, imbalanced bone remodeling, impaired BMSCs function, insufficient vascularization, and chronic inflammation. These factors collectively contribute to the poor regenerative capacity of osteoporotic bone, highlighting the need for targeted therapeutic strategies to address these multifaceted challenges.

### 2.2 Requirements of osteoporotic bone defects on repair materials

Current treatments for osteoporotic bone defects primarily include pharmacological interventions and surgical approaches. While drug therapies can alleviate symptoms to some extent, their efficacy in repairing bone defects is limited, and long-term use is often associated with adverse side effects (Black and Rosen, 2016). Surgical treatments, such as bone grafting and internal fixation, provide better mechanical support but are plagued by issues such as high graft resorption rates, increased infection risks, and the potential need for secondary surgeries (Reichert et al., 2009). The advent of bone tissue engineering has introduced innovative solutions for bone defect repair (Wang et al., 2023d; Wang et al., 2021b). However, traditional biomaterials mechanical mismatch with osteoporotic bone, failing to meet the specific repair requirements (Deininger et al., 2021). Therefore, scaffolds designed for osteoporotic bone defects must not only possess the biocompatibility, safety, and osteoinductive properties required for conventional bone repair but also meet more stringent criteria in terms of mechanical strength, osseointegration capability, and controlled drug release (Koons et al., 2020; Shuai et al., 2020; Zhang et al., 2020).

Above all, biocompatibility and safety require that the materials have no immune rejection, the degradation products are non-toxic, and possess cellular affinity. Prosthetic scaffolds need to mimic the matrix microenvironment of skeletal cells to promote adhesion, proliferation and differentiation of osteoblasts and BMSCs (He et al., 2020; Turnbull et al., 2018). Besides, the degradation rate of the prosthetic scaffold needs to be synchronized with bone regeneration, which requires controlled degradation of the material; otherwise, premature degradation will easily lead to scaffold collapse, and late degradation will hinder the growth of new bone into the scaffold. Most importantly, the significant reduction in the mechanical strength of osteoporotic bone requires a different mechanical fit for hydrogel scaffolds than conventional scaffolds (Silva and Gibson, 1997). Osteoporotic cancellous bone is 50%–90% less strong than normal, and cortical bone is 15%–25% less strong than normal (Jones et al., 2015). Therefore, the mechanical properties of hydrogel scaffolds need to match osteoporotic bone. Simultaneously, these scaffolds need to have adjustable stiffness and dynamic mechanical response, such as softness to support cell migration in the early stage of bone regeneration and enhanced stiffness to promote mineralization in the later stage. Furthermore, the therapeutic needs of osteoporosis impose strict requirements on the functional integration of hydrogel scaffolds. Hydrogel scaffolds need to have the ability to load anti-osteoporosis drugs (e.g., bisphosphonates, BP), growth factors, or bone morphogenetic proteins (BMP-2), and to achieve localized sustained release (Lei et al., 2023).

## 3 Overview of natural hydrogels

Hydrogels are cross-linked polymer chains with a three-dimensional (3D) network structure, which are capable of absorbing and retaining large amounts of water due to their hydrophilic groups, such as -NH_2_, -COOH, -OH, -CONH_2_, -CONH and -SO_3_H(14). Hydrogels have received much attention in the field of regeneration due to their structural similarity to extracellular matrices and their characteristics such as high water content, soft structure and porosity (Sun et al., 2012; Feng et al., 2023). In addition, hydrogels are widely used in biomedical applications such as wound healing, drug delivery, tissue engineering and 3D bioprinting, with strong multifunctionality and potential for clinical translation (Madaghiele et al., 2014; Zhong et al., 2021).

**FIGURE 1:**
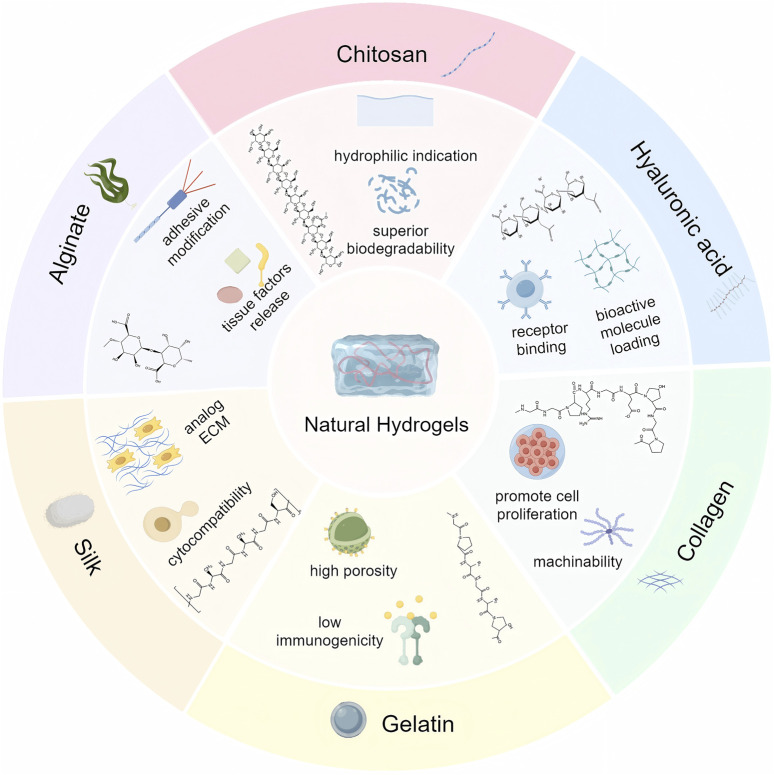
Natural hydrogels, with their chemical formulas and properties.

On the basis of the source of the polymer, hydrogels are mainly classified into natural hydrogels and synthetic hydrogels (Ho et al., 2022). Synthetic polymers used in synthetic hydrogels include poly (vinyl alcohol) (PVA), poly (ethylene glycol) (PEG), poly (ethylene oxide) (PEO), poly (2-hydroxyethyl methacrylate) (PHEMA) and poly (acrylic acid) (PAA) (Hu et al., 2022; Zhou et al., 2024; Li et al., 2022; Hakimi et al., 2023; Fatema et al., 2025). Natural polysaccharides include alginate, chitosan, hyaluronic acid (HA) and chondroitin sulphate, while natural polyamino acids include collagen, gelatin, silk, laminin, keratin, elastin, fibronectin, and heparin. Moreover, semipolymer hydrogels are a special type of hydrogel between natural and synthetic hydrogels, where the polymer is a chemically modified natural polymer or a combination of natural and synthetic polymers. In this article, they are included in modified natural hydrogels. Compared to synthetic hydrogels, natural polymer hydrogel scaffolds have better biocompatibility and lower immunogenicity in the treatment of bone defects (Table 1) (Yang et al., 2022). Common natural polymers are mainly polysaccharides and polyamino acids. Among them, the main natural polymers currently used for bone defect repair include alginate, chitosan, HA, collagen, gelatin and silk (Figure 1).

**TABLE 1:** Comparison of advantages and disadvantages of natural and synthetic hydrogels.

Features	Characterizations	Natural hydrogels	Synthetic hydrogels
Safety features	Biocompatibility	High (cell viability typically >90%)	Medium, requires surface modification
	Immunogenicity	Low immune rejection	High, requires surface modification to reduce risk of immune rejection
	Toxicity Risk	Degradation products are natural and non-toxic	May produce acidic or inert byproducts
Functional features	Mechanical Properties	Low (Young’s modulus of bare gelatin hydrogels is only 48 kPa) (Echave et al., 2022), need to be enhanced with inorganic materials	Wide range of tunability
	Degradation-regeneration synchronization	Degradation rate can be regulated by cross-linking degree	Difficult to control degradation, low synchronization
	Bioactive molecule loading	Naturally carries growth factor binding sites (hyaluronic acid binding BMP-2)	Requires chemical modification to introduce functional groups (e.g., methacrylate-loaded drugs)

BMP-2: bone morphogenetic proteins.

### 3.1 Polysaccharides

Alginate is derived from seaweed and consists of guluronic acid and mannuronic acid. There are advantages of good biocompatibility and non-antigenicity (Venkatesan et al., 2015). It is readily chemically modified with adhesion ligands and can control the release of tissue-inducing factors such as BMP-2. However, alginate is rarely used alone for bone regeneration due to the inability of mammalian cells to produce specific enzymes associated with degradation and the absence of its RGD sequence restricts cell attachment and reduces cytocompatibility. Therefore, alginates are usually compounded with other materials to obtain the desired functionality (Andersen et al., 2014; Zhang H. et al., 2021).

Chitosan is a class of polysaccharides existing in the shells of crustaceans such as crabs and insects (Huang et al., 2005). It possesses similar structure and properties to glycosaminoglycans, and its massive hydrophilic surface induces cell attachment, proliferation, differentiation, and migration. The β-(1,4) glycosidic bond between D-glucosamine and N-acetyl-D-glucosamine makes chitosan easy to be modified by chemical reactions, and it has excellent elasticity, flexibility, and less inflammatory response (Gu et al., 2013). Furthermore, the degradation products of chitosan are non-harmful aminosugars, completely absorbed in human body (Islam et al., 2020).

HA is a naturally occurring unbranched glycosaminoglycan with superior biocompatibility and biodegradability (Burdick and Prestwich, 2011). In particular, HA has remarkable hydrodynamic properties, especially in terms of viscosity and water retention capacity, which facilitate tissue homeostasis and biomechanical integrity (Zhao et al., 2014). It promotes bone formation and bone regeneration by binding to receptors (CD44 and RHAMM, etc.) expressed on skeletal cells and interacting with cytokines (Abatangelo et al., 2020). The mechanical properties of HA polymer chains can be modified by adjusting their degree of cross-linking and molecular weight for both load-bearing and flexible applications (Jeon et al., 2007). Moreover, HA has the potential to be incorporated into biologically active molecules through physical embedding, covalent immobilization, and other modifications to optimize the bioactivity and release kinetics of a wide range of biomolecules (Tiwari and Bahadur, 2019).

### 3.2 Poly-amino acids

Collagen is a naturally occurring acidic polymer of mucopolysaccharides commonly found in multicellular organisms, usually in the form of insoluble fibrous proteins, and is the main structural protein that makes up the walls of skin, cartilage, bone, and blood vessels, and a vital component of the extracellular matrix (Xu et al., 2021). With the advantages of lower antigenicity, greater porosity, greater hydrophilicity, and better biodegradability, it occupies a place in the field of bone defect repair (Lee et al., 2001). Chen et al. evaluated the healing ability between IDG-SW3 cells and BMSCs in OP mice using 3D collagen hydrogel scaffolds, confirming that collagen gels can provide a suitable environment for cell adhesion, proliferation and differentiation (Chen et al., 2020).

Gelatin is the product of partial hydrolysis of collagen and inherits most of the benefits of collagen while being less antigenic and easier to process and modify (Alipour et al., 2021). While inheriting most of the advantages of collagen, gelatin has lower antigenicity and temperature sensitivity (37°C to form gel), and is easy to be processed and modified. Furthermore, gelatin retains the Arg-Gly-Asp (RGD) sequence found in degraded collagen, which has the ability to promote cell proliferation and migration (Georgopoulou et al., 2018). Gelatine methacryloylamide (Gel-MA), a photosensitive biohydrogel material obtained by preparing methacrylic anhydride (MA) with gelatin, is the most prevalent form of gelatin used in bone tissue engineering. The formability of Gel-MA is far superior to that of collagen, and can effectively fill in the defective areas and provide a good 3D microenvironment for cell migration, adhesion and proliferation.

Silk consists of silk fibroin (SF, 70%) and silk gelatin protein (30%) (Mazurek et al., 2022). In particular, SF has excellent cytocompatibility, unique mechanical properties, non-toxic degradation products, controlled biodegradability, and ideal processability, and is extensively applicable to biomedical engineering applications, such as skin wound healing and repair, and bone/cartilage regeneration (Mazurek et al., 2022; Zhou et al., 2022). More importantly, SF serves as the fundamental part of composite scaffolds to mimic the ECM, which is an organic component of natural bone (Mottaghitalab et al., 2015). SF composite hydrogels can provide a biocompatible microenvironment for cell adhesion, proliferation, and osteogenic differentiation *in vitro*, promoting the formation of new bone and achieving successful repair (Cheng et al., 2021).

## 4 Modification strategies for natural hydrogels used to treat osteoporotic bone defects

Despite their inherent biocompatibility and biodegradability, natural hydrogels exhibit several limitations that hinder their direct application in bone tissue engineering, particularly for osteoporosis treatment. These limitations primarily include inadequate mechanical strength to withstand physiological loads and insufficient intrinsic bioactivity to effectively modulate the complex bone microenvironment. Furthermore, to address the specific pathological characteristics of osteoporosis, these hydrogels require additional functionalization to achieve multifunctional properties such as anti-inflammatory effects, pro-angiogenic capabilities, and inhibition of excessive bone resorption. To overcome these challenges and better mimic the structural and mechanical properties of native bone tissue while enhancing osteogenic potential, natural hydrogels are frequently modified through various strategies including chemical functionalization, composite formation with reinforcing materials, and incorporation of bioactive molecules or therapeutic agents.

### 4.1 Inorganic-organic composite scaffolds mimicking natural bone architecture

**FIGURE 2:**
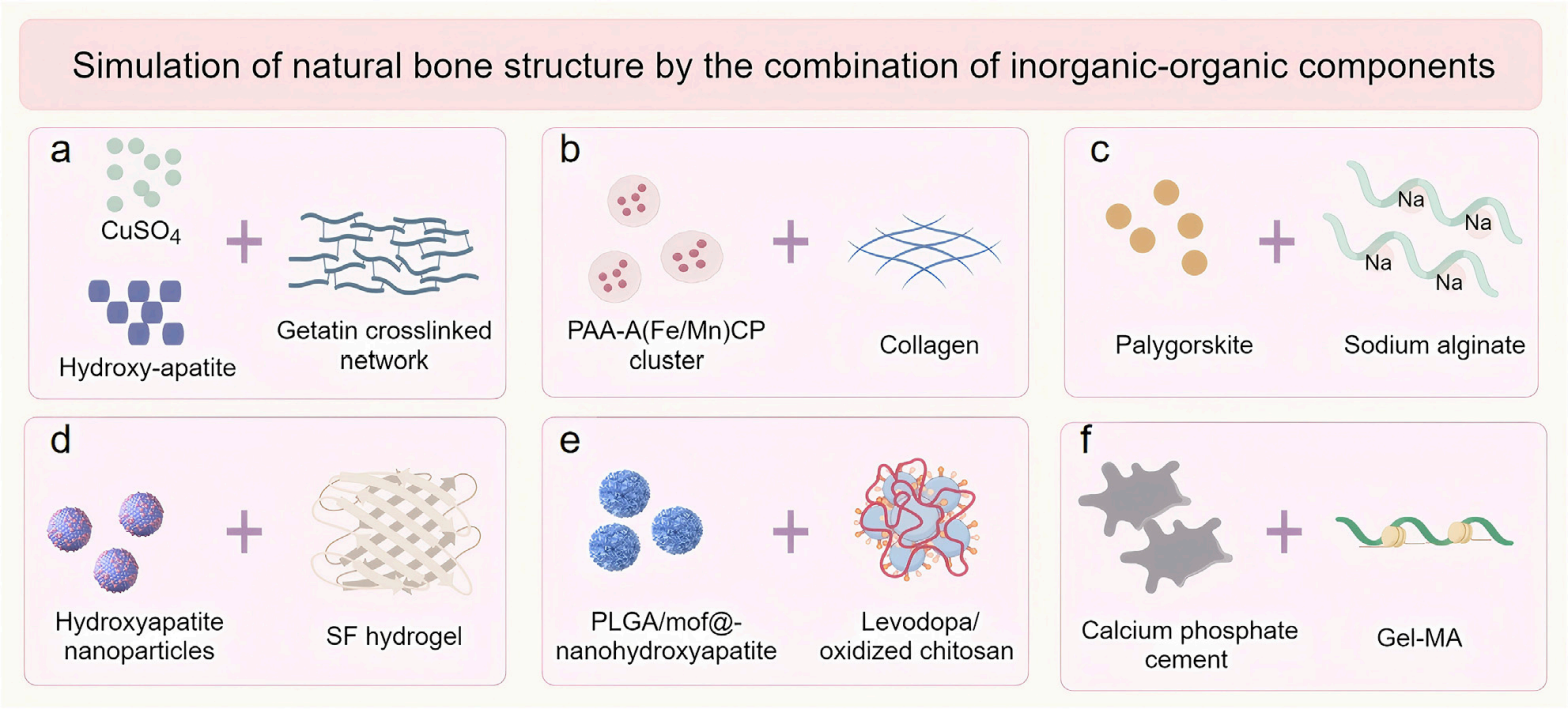
The inorganic-organic combination of modification strategies for natural hydrogels. **(a)** Hydroxyapatite, CuSO_4_ and Gelatin; **(b)** Hydroxyapatite bioceramics and Collagen; **(c)** Arygorskite and Alginate; **(d)** Hydroxyapatite nanoparticles and Silk fibroin (SF); **(e)** Hydroxyapatite and Chitosan; **(f)** Calcium phosphate cement (CPC) and Gelatine methacryloylamide (Gel-MA).

Natural bone tissue represents a sophisticated organic-inorganic composite system, comprising approximately 30% organic components and 70% inorganic hydroxyapatite (Yang J. et al., 2024). While natural hydrogel scaffolds demonstrate biocompatibility, their application in osteoporotic bone defect repair is often constrained by inadequate mechanical performance (Zhang Y. et al., 2021). The integration of inorganic materials with natural hydrogels has emerged as an effective strategy to enhance mechanical properties, achieving closer resemblance to native bone tissue (Figure 2). Commonly employed inorganic components include nanohydroxyapatite, calcium phosphate, silicon dioxide (SiO_2_), graphene oxide (GO), and bioactive glass (BAG) (Wang et al., 2021a; Zhao et al., 2016; Trementozzi et al., 2020; Zhai et al., 2015). These materials not only improve mechanical strength but also confer additional functionalities, such as enhanced osteoinductive capacity and drug delivery potential. For instance, nanohydroxyapatite provides essential calcium and phosphate ions that promote mineralization and scaffold rigidity, while BAG releases bioactive ions (Si^4+^, Ca^2+^) that stimulate osteogenesis-related gene expression (Trementozzi et al., 2020; Zhai et al., 2015).

**FIGURE 3:**
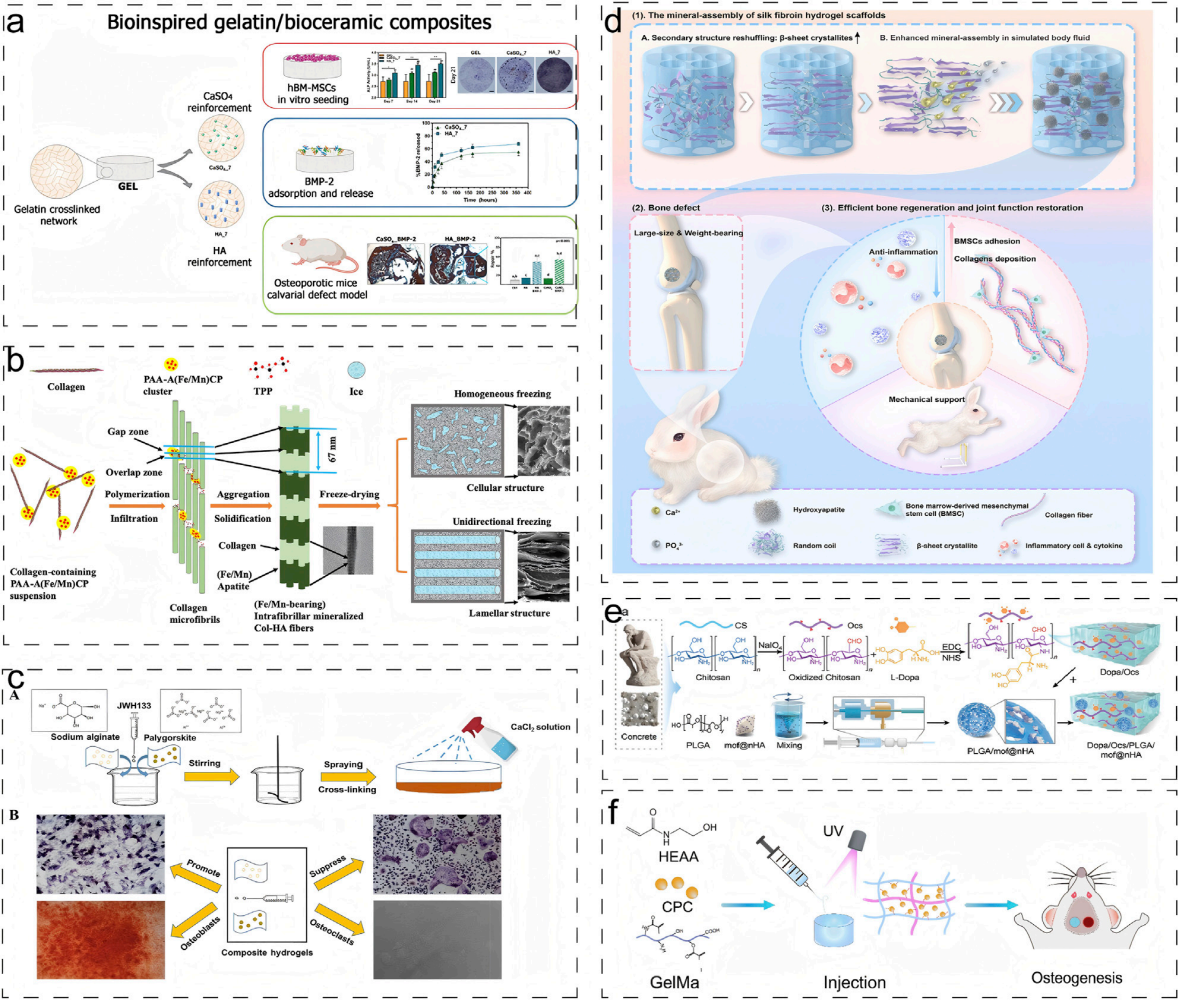
Engineering Natural Hydrogels via Combined Inorganic-Organic Modification: Strategies and Multifunctional Applications. **(a)** Schematic diagram of synthesis and application of biogelatin/bioceramic composite hydrogels loaded with BMP-2 (Echave et al., 2022). Reproduced with permission from ref. Echave et al. (2022). Copyright 2022 Elsevier. **(b)** Schematic illustration of the formation of intrafibrillar mineralized Fe/Mn-containing Col–HA scaffolds (Yu et al., 2020). Reproduced with permission from ref. Yu et al. (2020). Copyright 2020 American Chemical Society. **(c)** Schematic diagram of crosslinking technology to generate the composite hydrogel (Zhou et al., 2021). Reproduced with permission from ref. Zhou et al. (2021). Copyright 2021 American Chemical Society. **(d)** Schematic illustration of constructing mineral-assembled silk fibroin hydrogel scaffolds (Liang et al., 2024). Reproduced with permission from ref. Liang et al. (2024). **(e)** Schematic of the preparation of biomimetic bone glue inspired by concrete and its application in promoting the reconstruction of osteoporotic bone defects in the elderly (Li C. et al., 2024). Reproduced with permission from ref. Li C. et al. (2024). Copyright 2024 Wiley-VCH-GmbH. **(f)** Schematic representations of GelMA-PHEAA/CPC hydrogels for bone regeneration (Wang et al., 2023e). Reproduced with permission from ref. Wang et al. (2023e). Copyright 2023 MDPI, Basel, Switzerland.

Recent advancements in scaffold fabrication have demonstrated significant progress in this field. Echavarria et al. developed a three-dimensional hydrogel composite scaffold by incorporating hydroxyapatite bioceramics into an enzymatically crosslinked gelatin network (Figure 3a) (Echave et al., 2022). The incorporation of hydroxyapatite dramatically increased the Young’s modulus of the hydrogel from 48 kPa to more than 75 kPa, while decreasing the hydrolytic degradation rates. Similarly, Yu et al. engineered an innovative intrafibrillar mineralized Col-HA scaffold with cellular and laminar microstructures, incorporating essential micronutrients (Fe and Mn) that significantly enhanced bone repair outcomes (Figure 3b) (Yu et al., 2020). Zhou et al. demonstrated that the addition of Palygorskite (PAL) powder to alginate hydrogels improved both swelling capacity and mechanical properties, achieving a tensile modulus of 51.98 ± 4.76 kPa and compressive strength of 3.65 ± 0.30 MPa(Figure 3c) (Zhou et al., 2021).

Liang et al. pioneered an “organic-inorganic assembly” strategy to construct SF-based bone scaffolds with biomimetic mechanical properties (Figure 3d) (Liang et al., 2024). Through secondary structure reorganization, they achieved a 3.3-fold increase in β-sheet content, resulting in a 100-fold enhancement in mineral assembly efficiency and an increase in compression modulus to 2.33 MPa, which improves load-bearing bone regeneration. Li et al. developed a biomimetic bone gel incorporating poly (lactic acid-glycolic acid) microspheres within a chitosan hydrogel stabilized by nanohydroxyapatite (DOPM) (Figure 3e) (Li C. et al., 2024). It demonstrated exceptional mechanical properties including shear stress (1450 N), tensile strength (0.6171 ± 0.023 MPa), and adhesion strength (196.2 kPa). Wang et al. developed a fast crosslinking hydrogel system by combining calcium phosphate bone cement (CPC) with organic precursors gelatin methacrylate (Gel-MA) and hydroxyethyl acrylate (HEAA) (Figure 3f) (Wang et al., 2023e). The incorporation of the inorganic phase resulted in enhanced swelling stability and reduced degradation rate of the gel, while imparting excellent mechanical toughness to the hydrogel.

In summary, the incorporation of inorganic components into natural hydrogels presents a promising strategy for bone tissue engineering, as it closely mimics the organic-inorganic composition of native bone tissue while enhancing the mechanical strength of composite scaffolds. Moreover, this hybrid system not only improves osteoinductive potential but also facilitates controlled drug delivery. These synergistic effects position inorganic-organic composite scaffolds as a key modification strategy for treating osteoporotic bone defects. However, challenges such as material homogeneity, degradation kinetics, and biocompatibility must be carefully addressed during the design process.

### 4.2 Crosslinking strategies of natural hydrogels

Crosslinking strategies serve as crucial approaches for modulating the mechanical properties, degradation rates, and biological functions of natural hydrogels, primarily encompassing physical and chemical crosslinking methods (Xue et al., 2022). Physical crosslinking involves the formation of reversible three-dimensional networks through non-covalent interactions, such as hydrogen bonding, electrostatic interactions, and hydrophobic interactions, offering advantages of operational simplicity and mild reaction conditions (Wang et al., 2023f). In contrast, chemical crosslinking establishes stable three-dimensional networks via covalent bonds, significantly enhancing the mechanical strength and structural stability of hydrogels (Li B-H. et al., 2024). The common crosslinking strategies, along with their respective advantages, disadvantages, and specific applications, are summarized in Table 2. When individual crosslinking strategies prove insufficient for material synthesis requirements, composite crosslinking strategies combining multiple approaches are often employed to achieve desired properties.

**TABLE 2:** Characterization and application of common cross-linking strategies for natural hydrogels.

Typology	Specific strategies	Mechanism	Advantages	Disadvantages	Representative materials	Reference
Physial crosslinking	Temperature-induced crosslinking	Self-assembly of molecular chains induced by temperature changes	No chemical cross-linking agent required, suitable for *in situ* injection applications	Low mechanical strength, high temperature dependence	Gelatin	Segredo-Morales et al. (2018)
	Ionic crosslinking	Ionic bonding of anionic polysaccharides with multivalent cations	Fast cross-linking, gentle conditions	Poor gel stability, susceptible to ionic concentration	Alginate Chitosan	Kuo and Ma (2001)
	Self-Assembly Crosslinking	Self-assembly of hydrogen bonds or hydrophobic groups between molecular chains	Highly bionic, suitable for cell culture	Relatively weak mechanical properties	Collagen Gelatin	Aleksandr et al. (2024)
Chemical crosslinking	Schiff Base Crosslinking	Reaction of amino groups with aldehyde groups to form dynamic imine bonds	Reversible, self-repairing, pH-responsive	Poor long-term stability, easily degraded in acidic environment	Hyaluronic acid	Li et al. (2020)
	Photocrosslinking	Ultraviolet or visible light initiated radical polymerisation	Time and space controllable	Photoinitiators may cause cytotoxicity	Gelatin Hyaluronic Acid	Luo et al. (2024), Klara et al. (2024)
	Click Chemical Crosslinking	Covalent bond formation using biological orthogonal reactions	Efficient reaction, mild conditions	Requires chemical modification of natural macromolecules	Gelatin Hyaluronic Acid	Pan et al. (2020), Chen et al. (2023)
	Enzymatic Crosslinking	Use of enzymes to catalyse cross-linking reactions	Mild conditions, high biocompatibility	Expensive, crosslinking efficiency depends on enzyme activity	Chitosan	Cheng et al. (2022)

#### 4.2.1 Physical crosslinking modification

**FIGURE 4:**
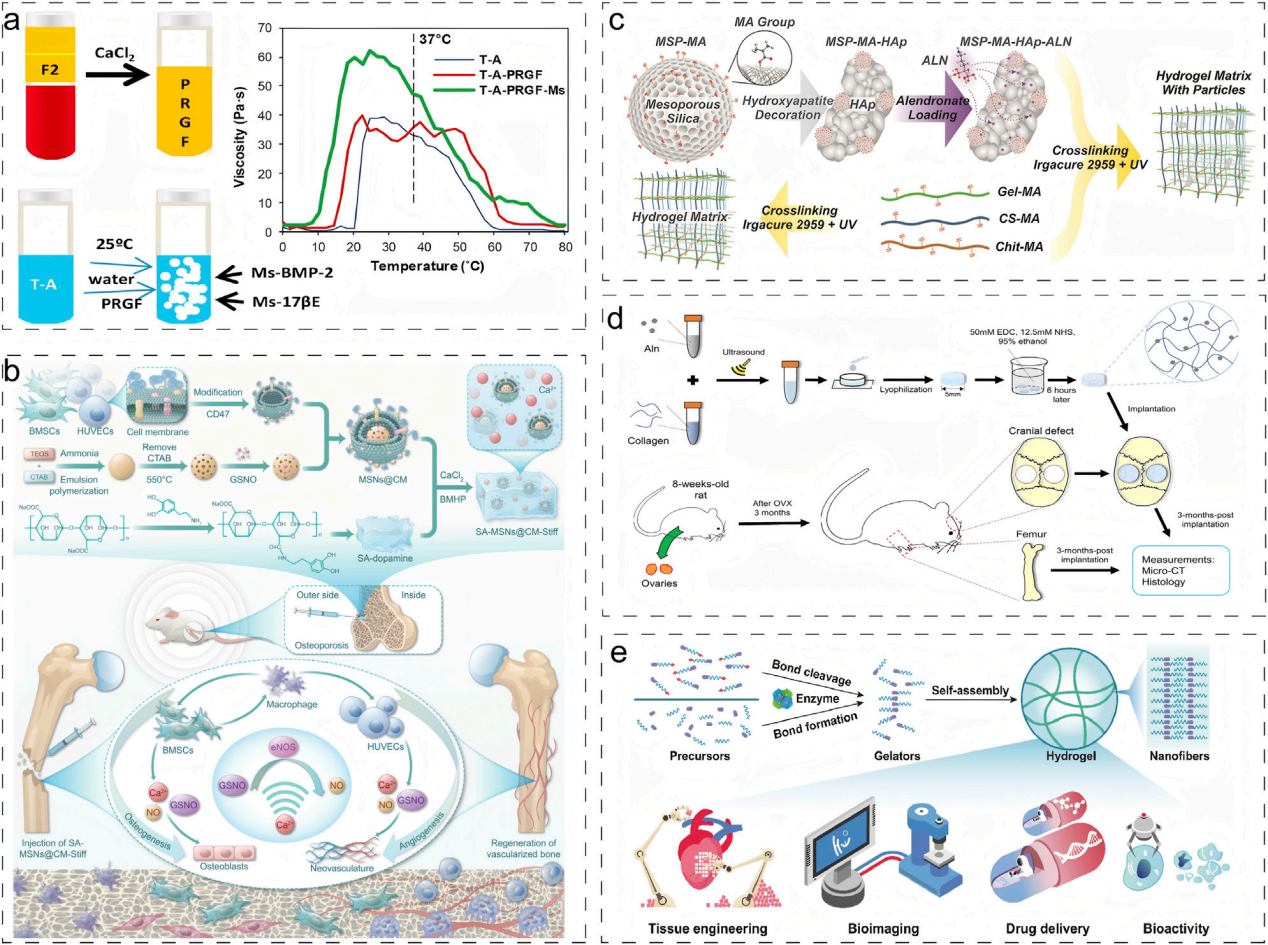
Typical application of physical and chemical crosslinking strategies for natural hydrogels. **(a)** Temperature-induced crosslinking strategy for physical crosslinking (Segredo-Morales et al., 2018). Reproduced with permission from ref. Segredo-Morales et al. (2018). Copyright 2018 Elsevier. **(b)** Ionic crosslinking strategy for physical crosslinking (Wei et al., 2024). Reproduced with permission from ref. Wei et al. (2024). Copyright 2024 American Association for the Advancement of Science. **(c)** Photocrosslinking strategy for chemical crosslinking (Klara et al., 2024). Reproduced with permission from ref. Klara et al. (2024). Copyright 2024 Elsevier. **(d)** Chemical crosslinking via condensation acylation reaction (Zeng et al., 2020). Reproduced with permission from ref. Zeng et al. (2020). Copyright 2020 John Wiley and Sons. **(e)** Enzymatic Crosslinking strategy (Cheng et al., 2022). Reproduced with permission from ref. Cheng et al. (2022). Copyright 2022 Elsevier.

Poloxamer, a thermoresponsive triblock copolymer composed of propylene oxide and ethylene oxide, undergoes reversible sol-gel transitions in response to temperature changes. Segredo-Morales et al. developed an innovative injectable thermo-responsive hydrogel scaffold by incorporating Poloxamer into alginate hydrogels, significantly enhancing scaffold stability (Figure 4a) (Segredo-Morales et al., 2018). In a separate advancement, Wei et al. engineered a sodium alginate (SA)-based hydrogel system through covalent conjugation with dopamine, improving cell-matrix interactions and cellular adhesion (Figure 4b) (Wei et al., 2024). By optimizing the calcium ion concentration to 50 mM, they achieved an enhanced elastic modulus of 25.56 ± 1.41 kPa, demonstrating improved mechanical properties.

#### 4.2.2 Chemical crosslinking modification

Klara et al. fabricated a multifunctional hydrogel system through a combination of photocrosslinking and freeze-drying techniques, utilizing methacryloyl gelatin (Gel-MA), chitosan, and chondroitin sulfate (Figure 4c) (Klara et al., 2024). This system allowed precise control over degradability and solubility by modulating biopolymer composition in the photocrosslinked network, thereby maintaining an optimal balance between impurity removal and new bone formation. Pan et al. designed a macroporous Gel-MA hydrogel incorporated with MgO nanoparticles via thiol-ene click chemistry (Pan et al., 2020). This innovative construct not only provided an ECM-mimicking microenvironment for *in situ* osteogenesis but also facilitated sustained release of Mg^2+^ ions, promoting osteogenic differentiation of BMSCs and accelerating bone regeneration. In another approach, Zeng et al. developed a sustained-release alendronate (ALN) delivery system through the formation of amide bonds between ALN’s primary amine groups and carboxyl groups in type I collagen, significantly enhancing the scaffold’s mechanical strength (Figure 4d) (Zeng et al., 2020).

Enzyme-mediated longitudinal hydrogelation through small molecule self-assembly represents a promising strategy for designing advanced functional biomaterials. Enzyme-initiated cross-linking approaches leverage endogenous enzymes to modulate cellular and tissue dynamics, offering significant potential for diverse biomedical applications, including drug delivery, tissue engineering, bioimaging, and *in situ* gelation (Figure 4e) (Cheng et al., 2022). Although enzymatic cross-linking strategies are increasingly recognized as valuable tools for modifying natural hydrogels, their application in addressing osteoporotic bone defects remains unexplored.

#### 4.2.3 Composite cross-linking modification

Ghavimi et al. demonstrated that incorporating neutral cellulose nanocrystals (CNCs) into dual-crosslinked chitosan hydrogels produced scaffolds with compressive strength (150–250 kPa) similar to that of osteoporotic vertebral bone, while simultaneously enhancing osteoinductive properties (Ghavimi et al., 2019). Graphene oxide (GO), with its abundant oxygen-containing functional groups, has shown remarkable potential for biomolecular crosslinking. Purohit et al. engineered a nanocomposite scaffold combining GO, gelatin, and alginate (Purohit et al., 2019). The scaffold not only supported MG-63 cell adhesion and proliferation but also allowed mechanical property modulation through GO concentration control. In a sophisticated approach, Gilarska et al. developed a hybrid hydrogel system by integrating apatite-modified silica particles (SiO_2_) conjugated with ALN into a collagen-based matrix containing lysine-functionalized hyaluronic acid and chitosan (ColChHAmod-Ap-ALN) (Gilarska et al., 2021). This multifunctional composite effectively replicated natural bone tissue in terms of both structure and composition, exhibiting superior physicochemical properties (including mechanical strength, wettability, and solubility) and achieving sustained ALN release.

### 4.3 Advanced modification strategies for natural hydrogels

#### 4.3.1 Metal ion-based functionalization strategies

**FIGURE 5:**
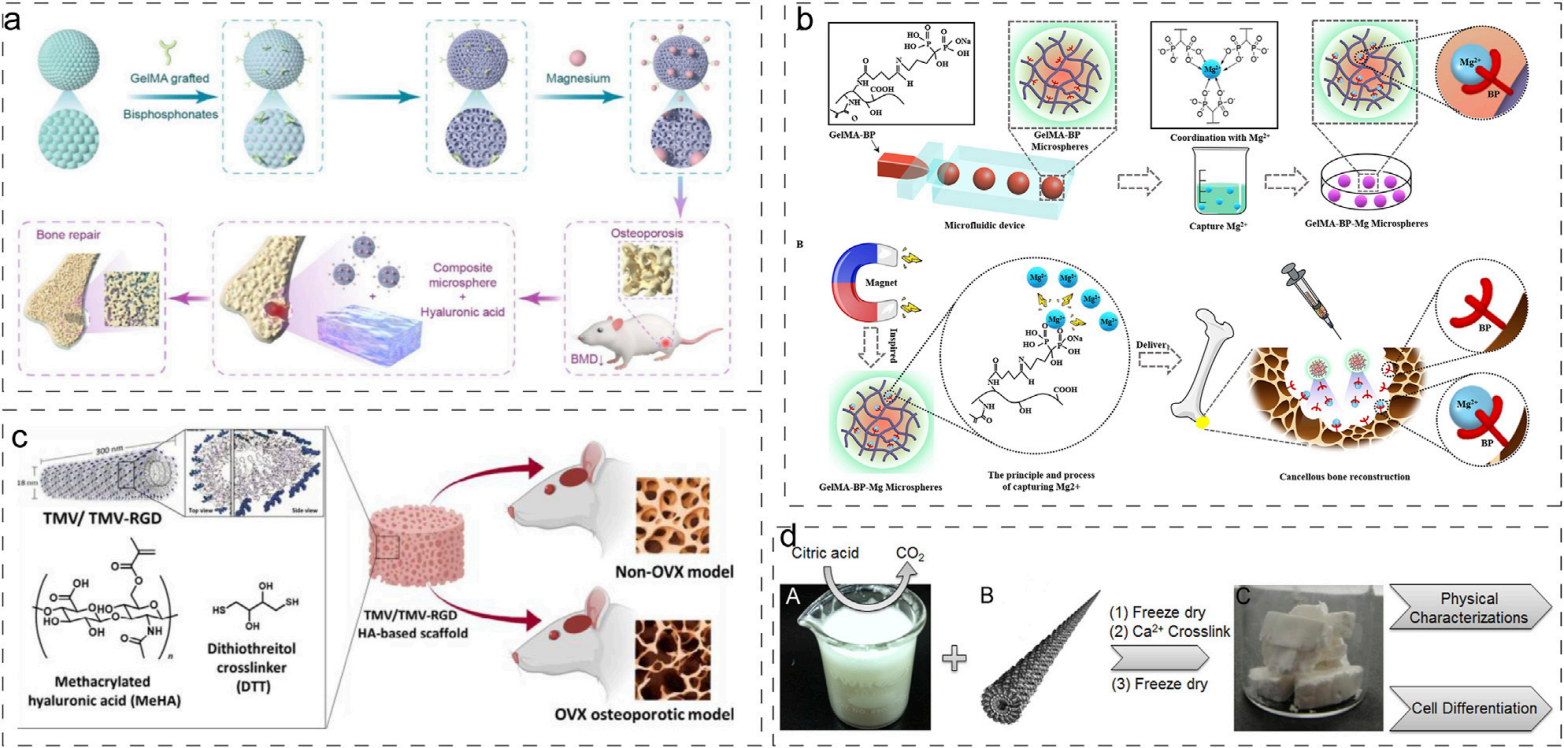
Advanced Modification Strategies for Natural Hydrogels. **(a)** Preparation process of IOHMs (Ma et al., 2024). Reproduced with permission from ref. Ma et al. (2024). Copyright 2024 Elsevier. **(b)** Preparation process of microfluidic GelMA-BP microspheres and construction of GelMA-BP-Mg microspheres that captured Mg^2+^ (Zhao et al., 2021). Reproduced with permission from ref. Zhao et al. (2021). Copyright 2021 American Chemical Society. **(c)** Synthetic procedure of generating virus functionalized porous composite hydrogels (Luckanagul et al., 2012). Reproduced with permission from ref. Luckanagul et al. (2012). Copyright 2012 American Chemical Society. **(d)** Synthesis of MeHA polymers crosslinked with TMV1cys via a sulfhydryl reaction (Yuan et al., 2020). Reproduced with permission from ref. Yuan et al. (2020). Copyright 2020 Biomaterials translational.

Ma et al. engineered an innovative multifunctional layered hydrogel system incorporating inverse opal methacrylated gelatin (Gel-MA) microspheres (IOHM-AS-Mgs) within a methacrylated hyaluronic acid (HAMA) hydrogel matrix, combined with alendronate sodium (AS) and Mg^2+^(Figure 5a) (Ma et al., 2024). This sophisticated design not only demonstrated enhanced cytocompatibility and superior cell adhesion properties but also significantly promoted osteogenic differentiation and vascular regeneration. In a parallel development, Zhao et al. fabricated BP-functionalized injectable hydrogel microspheres (Gel-MA-BP-Mg) through metal-ligand coordination chemistry (Figure 5b) (Zhao et al., 2021). This innovative system facilitated trabecular bone reconstruction in osteoporotic defects while enabling controlled release of BP through Mg^2+^ complexation.

#### 4.3.2 Biomolecular functionalization strategies

Jittima Luckanagul et al. developed a novel approach by incorporating engineered Tobacco mosaic virus (TMV) mutants (TMV-RGD1) displaying RGD adhesion peptides into sodium alginate hydrogel scaffolds (Figure 5c) (Luckanagul et al., 2012). This modification significantly enhanced cellular adhesion and viability within the hydrogel matrix. Building upon this work, they further advanced the technology by crosslinking methacrylated hyaluronic acid (MeHA) polymers with cysteine-modified TMV mutants (TMV1cys) through thiol-mediated reactions, enabling hydrogel formation under physiological conditions (Figure 5d) (Yuan et al., 2020). These functionalized hydrogels demonstrated excellent biocompatibility and significantly improved repair outcomes for both normal and osteoporotic bone defects.

## 5 Application strategies of natural hydrogels in osteoporotic bone defect treatment

**FIGURE 6:**
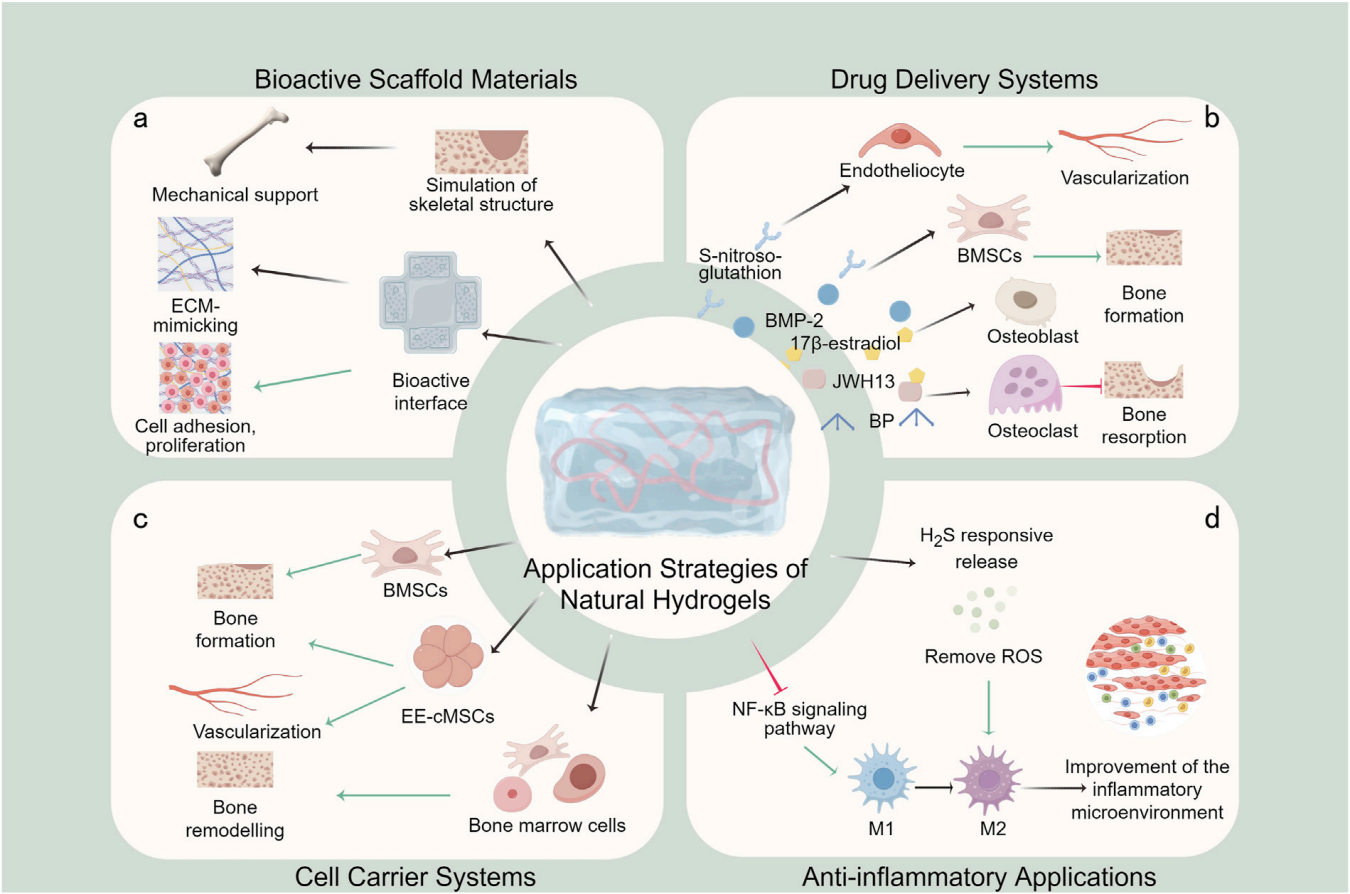
Application strategies of natural hydrogels in osteoporotic bone defect treatment. **(a)** Bioactive scaffold materials; **(b)** Drug delivery systems; **(c)** Cell carrier systems; **(d)** Anti-inflammatory applications.

Natural hydrogels have emerged as versatile platforms for osteoporotic bone defect treatment, primarily serving as bioactive scaffold materials, drug delivery systems, and cell carriers (Figure 6). Furthermore, considering the inflammatory bone microenvironment characteristic of osteoporosis, hydrogels are increasingly being designed for immunomodulation and microenvironmental remodeling, representing crucial therapeutic strategies.

### 5.1 Bioactive scaffold materials

The utilization of natural hydrogels as bioactive scaffold materials constitutes the most fundamental and widely adopted strategy in osteoporotic bone defect treatment (Figure 6a). Compared to other therapeutic strategies, natural hydrogels are highly biocompatible and structurally bionic and have the potential for minimally invasive applications. Its three-dimensional porous network structure can be precisely designed in terms of pore size (50–300 μm) and porosity (>80%) through the modulation of cross-linking density and material composition, and this optimized pore structure not only facilitates nutrient transport and metabolic waste discharge, but also provides a physical space for cell migration and vascular growth (Bruzauskaite et al., 2016). By strategically compounding with various materials, natural hydrogels can be engineered into three-dimensional scaffolds that modulate mechanical properties, pore structure and surface characteristics to optimize bone tissue regeneration. In particular, its inherent bioactive molecules (e.g., the RGD sequence of collagen, the CD44-binding domain of hyaluronic acid) can directly activate cell surface receptors and trigger osteogenesis-related signaling pathways (Ruoslahti and Pierschbacher, 1987; Kwon et al., 2019). These advanced composite scaffolds effectively mimic the composition and structural hierarchy of native bone tissue, providing mechanical support and a bioactive interface for tissue regeneration (Echave et al., 2022; Yu et al., 2020; Zhou et al., 2021; Liang et al., 2024; Yang W. et al., 2024; Miyazaki et al., 2015).

The bioactivity of these composite scaffolds extends beyond structural support, and it is due to the dual regulatory effects of physical structure and chemical signaling described above that they exhibit great potential in promoting adhesion, proliferation, and osteogenic differentiation of BMSCs (Echave et al., 2022). Among them, the micrometer-sized pores of the scaffolds promote cell spatial distribution and intercellular interactions, while the nanoscale fiber topology enhances cell stretching through contact-guided effects; meanwhile, the slow-released bioactive factors continuously activate the osteogenic pathways such as BMP/Smad and Wnt/β-catenin. In addition, they create a microenvironment that mimics the ECM, which greatly enhances bone repair in an osteoporotic mouse model.

### 5.2 Drug delivery systems

Natural hydrogels serve as excellent drug delivery platforms for osteoporotic bone defect treatment, capable of incorporating various therapeutic agents including anti-osteoporotic drugs (e.g., BP) and osteogenic factors (e.g., BMP-2) (Figure 6b). In contrast to other drug delivery systems, natural hydrogels allow for localised and sustained release of drugs from the defect site. Intelligent, stimuli-responsive release can also be achieved by adjusting cross-linking density or incorporating responsive molecules (e.g., pH-responsive, enzyme-responsive elements).

Van Houdt et al. developed a HABP-CaP hybrid nanocomposite gel via non-covalent crosslinking, combining hyaluronic acid, CaP nanoparticles, and BPs for controlled drug release and enhanced osteogenesis via BMP-2 (van Houdt et al., 2021). García-García et al. designed a sandwich-structured, approximately 72% porous biocomposite hydrogel loaded with BMP-2 and 17β-estradiol microspheres to achieve sustained dual-drug delivery of six and up to 6 weeks of release (Garcia-Garcia et al., 2019). Claudia Siverino et al. demonstrated the effectiveness of a thermo-responsive HA-pNIPAM hydrogel delivering zoledronic acid (ZOL) and BMP-2 (Siverino et al., 2024). In osteoporotic rat models, early (first 5 days) release of 80% zol from this hydrogel prevented early resorption and improved early stability, and sustained release of BMP-2 to enhance periprosthetic bone mass improved late stability. Zhou et al. expanded the cannabinoid receptor type 2 agonist JWH13 to alginate hydrogel scaffolds and achieved a sustained release of JWH13 for more than 7 days, effectively inhibiting osteoclast formation and enhancing osteoporotic bone repair (Zhou et al., 2021).

The application of natural hydrogels in drug delivery has expanded beyond conventional agents. Wei et al. developed a biomimetic hydrogel with stem cell homing capabilities, serving as a reservoir for S-nitrosoglutathione and Ca^2+^ (Wei et al., 2024). This innovative system promotes the release of bioactive nitric oxide (NO) from BMSCs and vascular endothelial cells, activating the NO/cyclic guanosine monophosphate signaling pathway to facilitate osteogenic-angiogenic coupling. Furthermore, the incorporation of the stem cell homing peptide SKPPGTSS enhances BMSCs recruitment, promoting bone tissue formation and neovascularization while exhibiting immunomodulatory properties.

### 5.3 Cell carrier systems

Compared to other carriers, natural hydrogels have excellent cytocompatibility and are excellent cell carriers by mimicking the natural ECM to provide cell adhesion sites, mechanotransduction signals and nutrient exchange channels. Natural hydrogels maintain cellular function by providing an optimal growth microenvironment that supports stem cell proliferation and promotes osteogenic differentiation (Figure 6c). These systems enable the encapsulation and localized delivery of mesenchymal stem cells (MSCs) or induced pluripotent stem cells (iPSCs), ensuring cell retention at the target site. While natural hydrogel-based cell carriers are primarily utilized to enhance bone formation, current research predominantly focuses on extensive bone defects, with limited specific applications for osteoporotic bone defects.

Teng et al. developed Gel-MA hydrogel microspheres (HMs) using a microfluidic system, in which BMSCs adhered to the HMs with a survival rate of up to 99% for 7 days without affecting the viability of the HMs, thus providing a platform for the adherence and proliferation of BMSCs to enhance the repair of bone defects (Teng et al., 2022). In another approach, Bastami et al. fabricated 3D-printed biodegradable hydrogels incorporating BMSCs within a matrix of alginate, gelatin, and lyophilized allogeneic bone nanoparticles (npFDBA) (Bastami et al., 2024). These 3D-printed constructs demonstrated superior BMSCs adhesion, proliferation, and differentiation capabilities, significantly promoting *in vivo* bone regeneration.

Notably, Yu et al. encapsulated human ectopic embryonic cranial-derived mesenchymal stem cells (EE-cMSCs) in a fibrin-collagen composite gel, which could maintain >90% cell viability (Yu et al., 2024). It effectively promoted bone remodelling and vascularisation after vertebral injury and targeted osteoporotic vertebral defects. In a separate study, Yu L et al. utilized a straightforward and practical strategy for bone repair by using Col-HA lamellar scaffolds loaded with fresh bone marrow cells mineralised within fibres to achieve sustained positive cell proliferation (Yu et al., 2020). Yang J et al. introduced an innovative post-bioprinting strategy to enhance the stability of hydrogel scaffold-loaded cells. Their approach involved loading cells into hollow hydrogel-based scaffolds (HHS) through a rapid, homogeneous, and precise method (Yang J. et al., 2024). The HHS demonstrated mechanical responsiveness to loaded cells within 4 s, with a 13-fold increase in cell loading capacity compared to static conditions, and cells exhibited uniform deposition and significant proliferation. This technique also enabled partitioned loading of two distinct cell types. The researchers validated the strong regenerative potential of HHS-loaded BMSCs in repairing osteoporotic bone defects in rat models.

### 5.4 Anti-inflammatory applications

Osteoporotic bone defects are characterized by abnormal inflammatory responses and a deteriorated bone microenvironment, where chronic inflammation driven by senescent macrophages leads to impaired repair and delayed healing. Consequently, anti-inflammatory strategies and local microenvironmental modulation are irreplaceable applications of natural hydrogels in osteoporosis treatment (Figure 6d).

Li et al. developed DOPM, a hydrogel system that promotes the polarization of senescent macrophages toward the M_2_ phenotype while inhibiting the NF-κB signaling pathway (Liang et al., 2023). This dual-action approach not only improves the inflammatory microenvironment but also enhances osteogenic differentiation, significantly accelerating bone defect repair and regeneration. In another innovative approach, Jiang et al. designed an injectable hydrogel capable of responsive release of H_2_S gas, effectively scavenging reactive oxygen species (ROS) and modulating macrophage polarization (Jiang et al., 2024). This system regulates the bone injury microenvironment, alleviates inflammation, and maintains osteogenic/osteoclastogenic balance, offering a promising therapeutic strategy for osteoporotic bone defects. Chen et al. developed an innovative composite hydrogel system by incorporating manganese dioxide (MnO_2_)-coated calcium phosphate microspheres (MMS) loaded with fibroblast-activated protein inhibitor (FAPi) into a methacrylated gelatin (GelMA) and methoxy polyethylene glycol (m-PEG) hybrid matrix. The composite hydrogel significantly reduced H_2_O_2_ and regulated macrophage polarisation, thereby improving the ROS microenvironment and inflammatory microenvironment of osteoporosis, ultimately promoting the repair of osteoporotic bone defects (Chen Q. et al., 2022).

## 6 Current challenges and future perspectives

### 6.1 Existing challenges

While bioactive materials offer innovative therapeutic strategies for osteoporotic bone defects, we regret to find that clinical studies and practical applications of natural hydrogels for osteoporotic bone defect treatment remain limited. The current evidence base is insufficient to draw definitive conclusions about their clinical efficacy and safety profiles. There are some clinical studies on the application of natural hydrogel in bone defects, but most of them are for alveolar and maxillofacial restorations, and have not been applied to osteoporotic bone defects (Alcantara et al., 2018; Kawai et al., 2017). Safety (e.g., immunogenicity) and cost-effectiveness (production, storage, and transport costs) are the main issues limiting clinical applications. Besides, the alteration of the hydrogel properties by the sterilisation process is another factor that needs to be controlled. Therefore, the majority of these innovative materials remain confined to animal experimentation stages, highlighting the substantial challenge of translating basic research findings into clinical applications.

Meanwhile, several challenges remain in the research of natural hydrogels because of the complex pathological environment of patients with osteoporotic bone defects. The primary challenge lies in achieving optimal synchronization between material degradation kinetics and bone regeneration rates (Yang et al., 2014). Current natural hydrogels often exhibit relatively rapid degradation profiles, which severely hinder bone regeneration processes. Addressing this issue requires the development of advanced real-time monitoring techniques to enable dynamic regulation of hydrogel degradation rates in response to the complex *in vivo* bone microenvironment. Fluorescent labelling systems and smart scaffolding materials that can quantify the efficiency of new bone formation are some examples (Huang et al., 2023). Additionally, existing animal testing protocols, with observation periods ranging from 4 to 8 weeks, fail to provide comprehensive data on the materials’ long-term performance and biocompatibility.

The development of smart, stimuli-responsive materials represents another significant challenge. These advanced materials, capable of responding to environmental changes in pH, temperature, or enzyme concentrations, hold great promise for osteoporosis treatment (Zhou et al., 2020). Specifically, stimuli-responsive drug-carrying hydrogels could potentially address unfavorable conditions in the osteoporotic bone microenvironment, including low pH, elevated reactive oxygen species (ROS) levels, and increased catabolic enzyme activity (Zhang et al., 2018; Wang et al., 2024c). However, the complexity of material modification and design has limited progress in this area, with controlled release of therapeutic agents and growth factors remaining suboptimal.

### 6.2 Future development directions

The development of multifunctional repair materials represents a crucial direction for future research (Xie et al., 2025). Future designs should incorporate multifunctional composites capable of providing mechanical support, controlled drug release, stem cell modulation, and comprehensive tissue repair to address the complex microenvironment and diverse patient needs. The development of spatio-temporal sequential release systems for micromultifunctional restorative materials provides the way forward. For example, multilayered core-shell structure hydrogels achieve rapid release of anti-inflammatory drugs and long-term release of anti-absorbent drugs (Ahmadi et al., 2020). Bidirectional cell-material regulatory systems are another potential approach for developing multifunctional materials. By integrating physical cues (e.g., construction of stiffness gradients), chemical cues (e.g., RGD peptide density optimisation) and biological cues (spatial patterning of ECM-derived peptides), cell fate and bone repair can be precisely regulated.

The development of smart responsive scaffolds is both a challenge and a necessary development for natural hydrogels. Inflammation-responsive drug release systems based on pH-sensitive bonds and ROS-sensitive units, and mechanically adaptive materials based on secondary cross-linking strategies (e.g., secondary photocross-linking) are currently the main directions (Huang et al., 2024). The emergence of CRISPR/dCas9 systems has opened new possibilities for developing gene-activated scaffolds (Liu et al., 2020; Zhong et al., 2024). This technology offers the potential for precise and sustained therapeutic effects through targeted regulation of osteogenesis-related genes, such as Runx2 and Osterix. Additionally, the integration of 4D printing technology with shape memory materials presents a promising avenue for developing smart hydrogel scaffolds (Chen X. et al., 2022). These advanced systems could enable dynamic adjustment of scaffold morphology in synchrony with the bone defect repair process, achieving superior tissue-scaffold integration.

## 7 Conclusion

Natural hydrogels, with their unique biological properties and functional versatility, provide a multidimensional approach to osteoporotic bone defect repair. By integrating controlled drug delivery, stem cell modulation, immune microenvironment remodeling, and advanced manufacturing technologies, these materials offer comprehensive solutions for bone regeneration. Future research must focus on addressing critical issues such as mechanical property optimization and precise matching of degradation-regeneration kinetics.

The development of next-generation hydrogel scaffolds featuring intelligent responsiveness and precise functional regulation holds the potential to overcome limitations of traditional treatments. These advancements could enable efficient and safe bone regeneration, ultimately facilitating the translation of hydrogel-based therapies from laboratory research to clinical applications. Through continued innovation and interdisciplinary collaboration, the field is poised to revolutionize osteoporosis treatment and bone defect repair strategies.
